# The Molecular Landscape and Utility of Multiomic Analyses in Triple-Negative Breast Cancer: Further Subtyping and Exploring Novel Biomarkers and Therapeutic Targets

**DOI:** 10.3390/cancers17244003

**Published:** 2025-12-16

**Authors:** Conan Juan, Yan Peng

**Affiliations:** 1 Department of Pathology, School of Medicine, Stanford University, Palo Alto, CA 94305, USA; 2 Department of Pathology, University of Texas Southwestern Medical Center, Dallas, TX 75390, USA; 3 Department of Pathology and Simmons Comprehensive Cancer Center, University of Texas Southwestern Medical Center, Dallas, TX 75390, USA

**Keywords:** triple-negative breast cancer, multiomics, tumor heterogeneity, biomarkers, therapeutic targets

## Abstract

Triple-negative breast cancer (TNBC) has the poorest prognosis among all breast cancer subtypes, largely due to the lack of targeted therapies and its resistance to both chemotherapy and immunotherapy. A deeper understanding of TNBC biology is therefore critical for identifying therapeutic targets. Molecular subtyping of TNBC, first introduced over a decade ago, has significantly advanced our knowledge of the disease’s biology. However, tumor heterogeneity remains a major factor contributing to poor clinical outcomes and treatment resistance. The integration of multiomic technologies, including genomic, transcriptomic, proteomic, and metabolomic analyses, offers a powerful approach to further dissect tumor heterogeneity and accelerate the discovery of new biomarkers and therapeutic targets. This review aims to highlight the potential utility and evolving role of multiomics (-omics) in improving our understanding of TNBC biology—particularly tumor heterogeneity—ultimately facilitating the development of novel therapies and actionable strategies to treat the disease.

## Simple Summary

Identification of targeted therapies and novel treatment strategies is critical for improving the survival of patients with triple-negative breast cancer (TNBC), which lacks ER, PR, and HER2 expression. Tumor resistance to chemotherapy and immunotherapy—the current systemic treatments for TNBC—significantly contributes to patient mortality. The biological complexity of TNBC, including its tumorigenesis, heterogeneity, and clinical behavior, remains insufficiently understood, despite the introduction of TNBC subtyping over a decade ago. Currently, multiomic technologies offer more powerful approaches to classify TNBC within a broad molecular landscape encompassing DNA, RNA, proteins, and other metabolites. These integrative analyses hold great promise for identifying novel biomarkers and actionable therapeutic targets to improve treatment outcomes. This review highlights recent advances in the use of multiomics for identifying targetable oncogenic drivers, predicting responses to neoadjuvant therapies, and discovering novel tumor biomarkers in TNBC.

## 1. Introduction

Despite advancements in diagnosis and treatment, breast cancer has an incidence of over 2 million worldwide and remains the leading cause of cancer mortality for women [1,2]. Triple-negative breast cancer (TNBC) refers to a heterogenous group of breast cancers that lack expression of ER, PR, and HER2. Despite recent advances with immunotherapy (IO) and antibody–drug conjugates (ADCs), TNBC remains the most aggressive subtype, characterized by a high risk of recurrence and a short overall survival in the metastatic setting [3,4]. As a result, there continues to be a need to identify biomarkers for diagnosis, prognosis, and treatment of these aggressive breast cancers.

Multiomics (also referred to as -omics) represent a potential avenue for interrogation of these novel gene targets. They are multimodal techniques that identify biological molecules (i.e., mRNA in the case of transcriptomics [5]) to gain a comprehensive understanding of biology and disease pathology. They generate tremendous amounts of data, which in the hands of skilled bioinformaticians can be used to comprehensively profile heterogeneous biologic samples. These techniques can be used to identify novel genes of interest from a background of hundreds if not thousands of genes, ultimately helping researchers work more efficiently to seek answers.

The purpose of this review is to explore current applications of -omics analyses and examine their potential role in the identification of novel biomarkers, with the goal of guiding the future of research on TNBC.

## 2. Multiomic Approaches to Cancer Research

Multiomic technologies can be used to identify mRNA (transcriptomics), DNA (genomics), protein (proteomics), metabolites (metabolomics), and lipids (lipidomics). Transcriptomic approaches explore expression levels of different genes on a sample-wide (bulk RNA-seq) or single-cell (scRNA-seq) level. Recent advancements have allowed these principles to be applied directly onto histologic sections to assess gene expression with spatial context (spatial transcriptomics). Table 1 contains an overview of some of these different -omics approaches, along with their strengths and weaknesses. Integrated approaches (e.g., single-cell RNA-sequencing combined with spatial transcriptomics) optimize each approach’s strengths to identify clinically relevant trends.

Multiomic techniques have been used to better understand the tumor biology of different cancers, from melanomas to gliomas and adenocarcinomas [26,27,28,29]. They have helped us to better understand the mechanisms behind checkpoint inhibitor response [30] as well as assess why some tumors are less responsive to immunotherapy [31]. They can also be used to monitor tumorigenic signaling between malignant and non-malignant cells [32]. In breast cancer specifically, they have been used to identify transcriptionally divergent subtypes of breast cancers [33] and to analyze samples of HER2+ tumor in conjunction with histology [34]. Furthermore, attempts have been made at using -omics to annotate HER2 status on histology [35] as well as to delineate the expression profiles of invasive ductal carcinoma and ductal carcinoma in situ [36]. Recently, they have been used for the identification of novel biomarkers for breast cancer diagnosis and treatment [37].

## 3. Multiomic Approaches in Triple-Negative Breast Cancer

Breast cancer has been classically divided into intrinsic subtypes based on expression of ER, PR, and HER2 in the PAM50 assay. These types include luminal A and B (both expressing hormone receptors, with luminal B expressing high Ki-67), HER2-enriched, and basal-like (which is primarily composed of TNBCs). These molecular subtypes of breast cancer have clinical implications on risk and survival [38,39].

Triple-negative breast cancer accounts for approximately 15% of breast cancers that lack expression of ER, PR, and HER2 [13]. They tend to be larger, higher in tumor grade, and more aggressive than other breast cancer types [3]. Because of their lack of hormone receptors or HER2 expression, no targeted therapies currently exist. Currently, treatment of TNBC relies on chemotherapy [13]. Only around 30% of TNBC cases respond to neoadjuvant chemotherapy [40], and even with successful chemotherapy, patients have higher risk of relapse and death after treatment [4]. Immunotherapies have demonstrated some effect, but some tumors are immune-cold and do not respond [41,42]. Although TNBC tumors are generally similar in their poor prognostic outcomes, there exists great heterogeneity between tumors—pathologically, mutationally, and transcriptionally [40]. Thus, there is a need to identify (1) prognostic indicators to stratify risk for TNBC cancers and (2) markers to target TNBC (and its different specific subtypes).

### 3.1. Triple-Negative Breast Cancer Molecular Subtypes Based on Gene Set Enrichment Analysis

Due to a lack of non-cytotoxic therapies, concerted efforts have been made to classify TNBCs molecularly for possible identification of novel treatment targets. Six primary subtypes, collectively known as TNBCtypes, were initially identified based on gene set enrichment analyses (GSEAs), comparing relative expression of different canonical pathways from microarray data [13,14,15,43]. These initial subtypes are summarized in Table 2. Interestingly, while TNBCs overall do not express ER or PR, the LAR subtype was enriched in pathways associated with hormone response. Since initial stratification, these TNBCtypes have been refined to four current subtypes (TNBCtype-4), with laser microdissection of histology revealing the original immunomodulatory (IM) and mesenchymal stem-like (MSL) groups to be non-tumor-associated cell lines.

The molecular TNBCtype-4 classifications have been shown to be clinically relevant [44], notably as predictors of complete pathologic response rates to neoadjuvant chemotherapy, with basal-like 1 (BL1) having the best response and basal-like 2 (BL2) and luminal androgen receptor (LAR) the worst [43,45]. Furthermore, multiomic investigations into these subtypes have revealed genes of interest that represent potential avenues of treatment for their associated subtypes (summarized in Table 3).

The classification of these molecular subtypes of TNBC based on GSEA has been a landmark finding; however, further studies reveal that TNBC complexity persists even past these subtypes. For example, while both “basal-like”, the BL1 and BL2 subtypes have notable differences that have been identified with further study. The BL2 subtype is enriched in genes that contribute to cancer invasion and motility [46], which could help explain why the BL2 subtype of TNBC have worse observed responses to treatment.

MicroRNAs, which are single-stranded, non-coding RNA that can post-transcriptionally regulate mRNA and cancer phenotype, represent an additional layer of complexity with these subtypes [48]. When compared with BL2, BL1 cancers have been demonstrated to upregulate the miR-17-92 cluster of microRNA [46], which is known to suppress E2F1 and PTEN, both of which are growth control proteins [49,50,51,52].

Furthermore, the LAR subtype of TNBC has been shown to exist on a spectrum. Distinct LAR subgroups (“sub-subtypes”) have been identified, with one being more biologically similar to mesenchymal TNBC and another being similar to basal TNBC [53]. Additionally, outside of these TNBCsubtype-4 analyses, comparisons of different TNBC groups have identified other markers that may help elucidate the mechanisms behind TNBC’s tumor heterogeneity.

### 3.2. Differentially Expressed Gene Analysis of Triple-Negative Breast Cancer

In order to better understand mechanisms behind TNBC growth and tumorigenesis, research has been conducted comparing different phenotypes of TNBC to find differentially expressed genes (DEGs). DEG analysis is a method of identifying potential genes of interest when comparing different populations of study [54]. Out of hundreds if not thousands of genes, it identifies genes of interest that are over-expressed or under-expressed in one population versus another. Comparisons can be inter-sample (between separate biological samples, i.e., comparing expression profiles between cancers with different TNM staging) or intra-sample (within the same sample, i.e., comparing different cell populations within the same tumor) [55].

The fundamental idea behind DEG analysis is that differences in gene expression ultimately contribute to differences in observed phenotype—whether that be disease aggressiveness or treatment response. Identification of these DEGs can be a vital step in understanding how different disease pathologies come to be and can be helpful when exploring phenomena that are incredibly complex and driven by a variety of factors. In the case of triple-negative breast cancer, DEG analysis can be performed with -omics techniques to investigate genes which underlie differences in prognosis and treatment response.

#### 3.2.1. Immune-Related Genes

The cancer-immunity cycle, where the innate immune system catalyzes the adaptive response to tumor antigens, defines the body’s response to cancers [56]. Mechanisms to evade this system [57] allow cancer cells to proliferate and tumors to become malignant. Yet, even when the immune system is unable to prevent cancer development, the advent of immunotherapies, like checkpoint inhibitors and monoclonal antibodies, have proven effective at harnessing the immune system’s natural capabilities to treat cancer [58]. For example, in high-risk, early-stage TNBC, the immune checkpoint inhibitor pembrolizumab has achieved significant improvements in pathologic complete response [59,60]. In advanced metastatic disease with positive PD-L1 expression, pembrolizumab has also demonstrated significant effects on survival [61] and has become standard of care. Despite these advancements in treatment, TNBCs are heterogeneous, and a subset have molecular profiles that in some way impact their ability to respond to immunotherapy. -Omics techniques utilizing differential analysis have been used to identify key players in this chain, from immune response to immune therapy.

To evaluate the spectrum of immune reactions to TNBC development, differential gene expression analysis can be used to compare TNBC samples of different severities. These analyses help identify novel markers for downstream analysis. For example, macrophage inhibitory factor (MIF) is secreted by cancer cells and has been thought to drive immune evasion [17,18,19]. In Chen et al., RNA-sequencing across TNBC samples of varying severity revealed differential expression of MIF, with higher expression of this gene correlating with a higher T stage. Furthermore, through the integration of other -omics approaches, Chen et al. were able to implicate a possible mechanism underlying this finding. Single-cell RNA-sequencing analysis, which can be used to infer cell-to-cell interactions, identified epithelial breast cancer cells which secrete MIF, potentially mediating the polarization of macrophages into the M2 phenotype. Spatial transcriptomics corroborated this result, with epithelial cell markers and MIF expression found to be expressed in concurrent regions [16].

Even with the adoption of novel immunotherapies, TNBC has proven to be a formidable foe, with many cases of TNBC being non-responsive to immune checkpoint inhibitors [41,42]. Much like with tumor severity, differential analysis of -omics data can be performed to better understand the genes that are involved in this phenomenon. In Liu et al., analysis of single-cell sequencing data from both immune checkpoint responders and non-responders revealed SIGLEC9 (in myeloid cells) and TNFSF9 (in dendritic cells) to be highly expressed in non-responders compared to responders [62]. Interestingly, -omics techniques have similarly implicated SIGLEC9-expressing macrophages in other cancers that fail to respond to anti-PD-1 therapy [63], as well as TNFSF9 in metastatic progression [64], altogether suggesting that these two genes somehow contribute to immune-cold responses to immunotherapy in TNBC. On the other hand, investigations of immunotherapy response have also identified genes that could be important predictors of a successful response to immune checkpoint inhibitors. In Zhang et al.’s comparison of single-cell RNA-sequencing from responders and non-responders, CXCL13 expressed in T cells was found to be enriched in responders prior to treatment. Additionally, for responders who received a combination of chemo- and immunotherapy, the population of CXCL13-expressing T cells significantly expanded from baseline. This pattern was not mimicked in patients who responded to monotherapy paclitaxel, who interestingly exhibited decreased levels of CXCL13+ T cells after treatment. These observations in single-cell sequencing suggest that not only is CXCL13 significant for TNBC response to immunotherapy, but the addition of paclitaxel with these treatments may actually inhibit the expansion of CXCL13+ cells and dampen the effect of immune checkpoint blockers [65].

Differential analysis of gene expression and protein translation can be further applied to the intrinsic subtypes of breast cancer to explore immune therapy response. In Zhu et al., differential analysis of proteomics comparing all intrinsic breast cancer subtypes revealed CD73, a protein which inhibits immune responses, to be enriched in TNBC. To explain this finding, the authors implicated OTUD4, which stabilizes CD73 [11] in TNBC cells, causing its eventual accumulation and downstream immunosuppressive effects. With additional spatial transcriptomic analysis, OTUD4 and CD73 were found to be positively correlated in malignant regions across samples of TNBC [12]. Here, -omics techniques allowed the authors to rapidly profile hundreds of proteins to identify a potential protein for further investigation. Armed with this knowledge, Zhu et al. investigated possible treatment modalities targeting the OTUD4/CD73 axis by developing a pharmacologic inhibitor of OTUD4, ST80, which restored T cell function and increased the efficacy of immune checkpoint therapy [12].

#### 3.2.2. Epithelial Cells

The principles of differential gene expression have also been applied to the epithelial cells that make up TNBC tumors. Through single-cell RNA-sequencing, Kim et al. sought to establish markers that separate TNBC epithelial cells from other breast cancers. Using this method, the authors identified 12 DEGs which were highly expressed in TNBC epithelial cells compared to normal breast tissue, ER+, and HER2+ breast cancer [8]. These included multiple genes that are involved in epithelial to mesenchymal transition: vimentin (VIM) and caldesmon (CALD1).

In epithelial cell cancers (carcinomas), the epithelial to mesenchymal transition has been heavily implicated in cancer progression and metastasis. During this process, epithelial cells shed their epithelial characteristics in favor of upregulating mesenchymal cell markers. Under typical conditions, this transition contributes to wound healing; however, in carcinomas, it increases cellular motility and allows epithelial cell cancers to become invasive [66]. Furthermore, genes involved in this process have been associated with drug resistance [67], underlying the importance of studying these genes through techniques like -omics.

VIM, an intermediate filament protein found in mesenchymal cells [68], is required for the epithelial to mesenchymal transition and has been shown to promote TNBC invasion [69,70]. Silencing of the estrogen receptor leads to the development of the vimentin cytoskeleton [71], which is believed to be one of the driving factors behind the aggressiveness of TNBC. Furthermore, pre-clinical experiments assessing the effect of vimentin knockdown on tumorgenicity have exhibited decreased metastatic progression, suggesting the possibility for VIM not only as a marker of TNBC, but also for future therapeutic investigation as well [70].

CALD1 encodes caldesmon, an actin-binding protein that regulates cell motility via actomyosin systems in both muscle and non-muscle cells [72,73]. Caldesmon has previously been implicated in other carcinomas, including colorectal cancer [72,74]. In breast cancer cells, silencing of the estrogen receptor led to CALD1 upregulation [71]. Further studies of CALD1 in breast cancer have suggested that CALD1 could be a marker for tamoxifen resistance and cancer recurrence [75].

In sum, applying differential gene expression analysis to data from -omics techniques has identified many markers of TNBC—some associated with better or worse treatment response, cancer severity, or prognosis. These markers are shown in Table 4 with their current functional validation status.

### 3.3. Metabolomic Investigations of Triple-Negative Breast Cancer

In metabolomics, mass spectroscopy is used to comprehensively profile a wide breadth of small molecules, including amino acids, lipids, and sugars [77]. Major changes can be seen in individual metabolomes after relatively small stimuli, giving metabolomics potential for early detection of diseases, including triple-negative breast cancer [24].

Metabolomics have been used to better understand TNBC evasion of normal cell death mechanisms. Ferroptosis is a non-apoptotic form of cell death that is driven by iron and the peroxidation of lipids [78]. In the LAR subtype of TNBC specifically, Yang et al. used -omics to identify glutathione metabolism as a key pathway for ferroptosis evasion by cancer cells. Metabolomic analysis showed that glutathione-related metabolites like L-glutamate and glycine were significantly enriched in LAR cancers. Furthermore, by additionally utilizing scRNA-seq, Yang et al. showed increased expression of genes involved in glutathione metabolism in androgen receptor-expressing TNBCs [25].

Like the other -omics techniques, metabolomics can be used to stratify TNBCs into smaller groups based on differential metabolite profiles. Gong et al. used metabolomics on 72 samples of TNBC, in addition to GSEA with transcriptomic data, to identify three metabolically defined subtypes of TNBC: MPS1 (with upregulated lipid metabolism), MPS2 (with upregulated carbohydrate/nucleotide metabolism), and MPS3 (with mixed pathway dysregulation). With these groups in mind, the authors tested metabolic inhibitors on cancer cell growth and found that MPS1 TNBC cells were sensitive to inhibitors targeting lipid pathways and MPS2 TNBC cells were sensitive to inhibitors targeting glycolysis [79].

Similarly, Xiao et al. identified three metabolic subtypes of TNBC in their atlas of 330 TNBCs and 149 paired normal breast samples: C1 (enriched in fatty acids and ceramides), C2 (enriched in metabolites associated with oxidation reaction and glycosyl transfer), and C3 (group with the lowest level of pathway dysregulation). Using transcriptional analysis, they found that the metabolically defined C1 subtype heavily overlaps with the LAR subtype of TNBC, leading them to test the efficacy of ceramide inhibitors on LAR tumors. Notably, fingolimod, an FDA-approved drug targeting the ceramide pathway, was effective when used in LAR cells, suggesting a possible subtype-specific TNBC target [80].

### 3.4. Epigenetic Findings in Triple-Negative Breast Cancer

Epigenetic modifications can alter the state of DNA (e.g., heterochromatin vs. euchromatin) or post-translationally change amino acids. They affect biologic phenotype without mutating underlying DNA. Dysregulation of the epigenome is common in tumor biology [81], making it a growing field of interest in cancer research.

Epigenetic interactions can help explain immune evasion in TNBCs. From RNA-seq data, Chen et al. identified AT-rich interaction domain 1A (ARID1A), an epigenetic modifier, in TNBCs which displayed adaptive immune resistance. Low levels of ARID1A were linked to poor outcomes via adaptive immune resistance and increased expression of PD-L1. However, ARID1A itself did not regulate PD-L1 levels. Instead, it mediated PD-L1 levels via the transcription regulator, NPM1. Using ATAC-seq, which assesses chromatin accessibility [21], they demonstrated that low levels of ARID1A lead to increased chromatin accessibility of NPM1, which in turn upregulates PD-L1 expression [23] and ultimately results in suppressed T cell activity [82]. With this discovery in mind, the authors looked at the results and tissue samples from the CTR20191353 clinical trial, which tested pucotenlimab, a humanized anti-PD-1 antagonist [83]. Patients with tumors that were low in ARID1A demonstrated significant improvements in progression-free survival, suggesting that while low levels of ARID1A contribute to immune evasion in TNBCs, they may render tumors more responsive to immune checkpoint inhibitors [23].

## 4. Discussion

**Figure 1:**
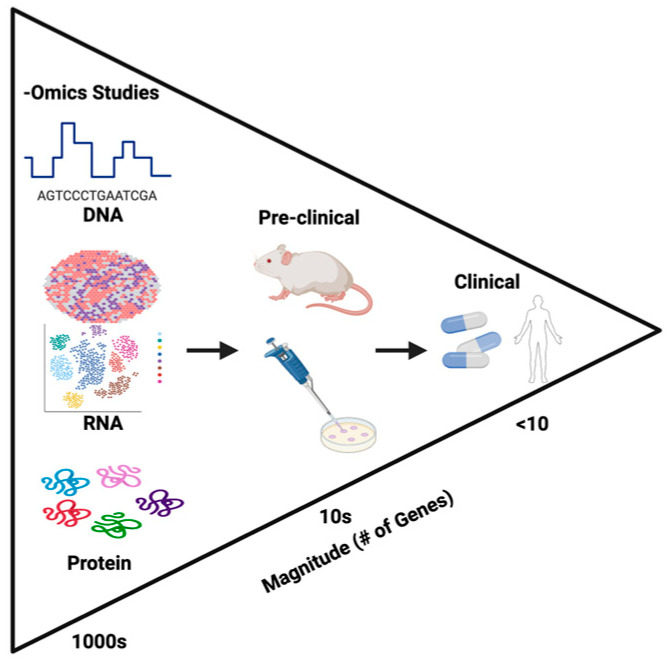
Proposed pipeline using -omics techniques to identify biomarkers in TNBC. Multiomic techniques enable investigators to draw information from multiple bioinformatics sources, identifying a specific set of genes of interest, for use in more labor- and time-intensive downstream models.

Because each of the multiomic approaches have their own strengths and weaknesses, the most compelling of research studies integrate multiple approaches in their investigations of triple-negative breast cancer. Many studies [12,16,23,80] draw upon multiple -omics techniques to help guide investigators to their final conclusions. As in Xiao et al.’s epigenetic findings with ARID1A [23], often one -omics technique can be used to profile and identify initial genes of interest (in their case, transcriptomic analysis first identified ARID1A as an epigenetic modifier of interest). These preliminary findings allow for hypothesis-driven downstream analysis, where additional -omics techniques (i.e., ATAC-seq in the case of Xiao et al.) can then be used to further explore and elucidate mechanisms underlying their initial observations. Such combined approaches can be used to profile hundreds if not thousands of genes in order to identify potential novel biomarkers of TNBC. These isolated genes of interest can then be further validated pre-clinically using in vitro cell lines and animal models. Finally, the most promising of these genes can finally be evaluated clinically to explore potential new avenues of TNBC classification, risk stratification, and treatment. This proposed pipeline of -omics to bedside investigation is illustrated in Figure 1.

While the downstream effects of -omics implementation in TNBC research have yet to be fully realized, these techniques have already advanced our understanding of TNBC. For instance, the discovery of the TNBCtype-4 subtypes of triple-negative breast cancer has led to investigations of new targeted therapies, exploring individualized therapies against genes enriched in each of the molecular subtypes of TNBC [84]. The knowledge that basal-like TNBCs upregulate growth factor genes has led to investigations into the efficacy of targeting epidermal growth factor receptor (EGFR) [85,86], vascular endothelial growth factor receptor (VEGFR) [87,88], and fibroblast growth factor receptor (FGFR) [44,89]. Furthermore, the identification of the LAR subtype has driven research into androgen receptor targeting [44] as well as into the effects of PI3K inhibitors [84], which have been shown to make cancers more susceptible to DNA-damaging agents [90].

The TNBCtype-4 subtypes of triple-negative breast cancer have also demonstrated some potential for stratifying responses to chemotherapy regimens. For instance, the p53 family of tumor suppressors has been shown to be associated with the basal-like classes of TNBC, suggesting its status as a potential predictive marker for response to paclitaxel/cisplatin with everolimus [84].

Further applications of -omics techniques using differential gene analysis have identified key genes that distinguish TNBC from other types of breast cancer. These analyses can also identify how different subtypes (and even sub-subtypes) of TNBC differ from each other, to help us understand the heterogeneity that underlies all the different phenotypes of TNBC that are observed. However, because of this complexity, true understanding of TNBC remains elusive. Even within the TNBCsubtype-4 classifications, sub-subtypes exist. Additional markers that have been discovered independently of the TNBCsubtype-4 classifications are further evidence of the deep heterogeneity of TNBCs. It is unlikely that a “one size fits all” or even a “one size fits most” approach is possible with TNBC—as treatment responses have been demonstrated to be multifactorial and not wholly dependent on any single known marker, an idea that has been exemplified with programmed death ligand 1 (PD-L1). The advent of immunotherapies has been an exciting advancement in oncology, as immunotherapies like atezolizumab and pembrolizumab can be used in a targeted fashion against PD-L1/PD-1. In the case of TNBC, however, PD-L1 status is a poor predictor of tumor response to atezolizumab with carboplatin. Although the drug directly targets PD-L1, certain subtypes of TNBC can respond to immunotherapy regardless of their PD-L1 status, suggesting that this one gene is not capable of stratifying TNBC into responders and non-responders [91].

It is clear that triple-negative breast cancers have vast complexity. In spite of the nature of the disease, -omics techniques have incredible potential to tackle this challenge. In particular, spatially resolved -omics technologies enable researchers to understand TNBC tumor cells in the context of their microenvironment, providing insights into cell-to-cell interactions, immune infiltration, and stromal contributions to tumor behavior. Advancements and further applications of -omics, like the advent of sub-cellular resolution for spatial transcriptomics with the Xenium platform [92] or the profiling of non-coding sequences like microRNA [93] or long non-coding mRNA [94], make the field even more exciting.

## 5. Future Directions to Enhance the Clinical Applicability of -Omics in TNBC

-Omics have proven their worth in basic and translational research, helping us understand tumor biology and heterogeneity and discover novel targets for the treatment of TNBC. Still, the ultimate goal for these techniques is to be integrated into routine clinical workflows for cancer management. Challenges exist to integrating -omics into clinical practice. These techniques generate immense amounts of data, which require both the knowledge and time to leverage. Furthermore, cost remains a concern for multiomic studies (the techniques themselves are often costly to run even without considering labor costs). However, as with any new and emerging technology, the expenses will continue to decline as more innovations are made to improve these techniques. Despite the challenges, we remain hopeful that -omics techniques and principles will be applicable to clinical care. Specifically, future clinical uses of -omics may include the following:

### 5.1. Comprehensive Molecular Profiling

Continued development of high-resolution -omics platforms will enable more accurate prediction of gene expression patterns, facilitating the identification of therapeutically targetable oncogenic drivers in TNBC. This will support more precise and personalized treatment decision-making.

### 5.2. Therapy Response Prediction

With improved sensitivity and analytical power, -omics-based approaches could more reliably predict responses to neoadjuvant treatments, including chemotherapy and anti-HER2-directed therapies. Importantly, optimization of protocols for small-volume specimens, such as core biopsies or fine-needle aspiration samples, may further enhance clinical feasibility.

### 5.3. Biomarker Discovery and Validation

-Omics technologies hold strong potential for the discovery of novel diagnostic, prognostic, and predictive biomarkers. Future efforts should focus on validating these biomarkers in large, multi-center cohorts to accelerate their translation into clinical practice.

## 6. Conclusions

Triple-negative breast cancer remains one of the most complex and poorly understood cancers to afflict humanity. As such, there is a need to continue exploring novel biomarkers for the classification and treatment of TNBC. Multiomics (or -omics) are multimodal techniques that are used to identify, profile, and understand biological molecules. These molecules drive the mechanisms behind disease, including TNBC. In the hands of skilled bioinformaticians, -omics can be used to tackle the complexity of TNBC in a way that was not previously possible. These techniques can be leveraged to explore thousands of genes between multiple experimental conditions to better understand TNBC and identify novel biomarkers for further validation in pre-clinical and clinical trials. As these technologies continue to advance, they should be further integrated as tools used to study and classify TNBC.

## Figures and Tables

**Table 1:** Overview of multiomic approaches.

Technique	Description	Strength	Weakness	Example Findings
Single-cell transcriptomics (scRNA-seq)	Cellular level analysis of mRNA expression	High resolution. Identifies cell populations in heterogeneous samples [6].	Cost [7]. Lacks spatial context.	VIM, CALD1 [8]
Spatial transcriptomics	Analysis of mRNA from sample on slide	Shows trends with spatial context. Probe sets for the entire transcriptome are available. Has promising future as more advanced products are released.	Cost [9]. Low resolution. Relatively low transcript capture in certain tissue types (e.g., mineralized tissue [10]).	CD73/OTUD4 [11,12]
Microarray gene expression analysis	Collection of mRNA using array of known probes	Used for targeted studies.	Can only identify expression of known probes.	TNBCtype [13,14,15]
Bulk RNA-seq	Analysis of mRNA from whole sample	More sensitive than microarray and does not require specific probes.	Primarily for broad sample-wide trends.	MIF [16,17,18,19]
Proteomics	Analysis of protein structure and function	Clinically relevant protein identification. Cost-effective methods [20].	Complexity of protein structure. Difficulty for studying post-translational modifications.	CD73/OTUD4 [11,12]
ATAC-seq	Assessment of chromatin accessibility utilizing transposase-tagged DNA	Explores the impact of epigenetic modifications on observed phenotypes.	Biases from transposase reaction, abundance of mitochondrial reads [21,22].	ARID1A [23]
Metabolomics	Mass spectroscopy separates metabolites into component parts for downstream analysis	Contribution of small molecules (i.e., lipids, amino acids, sugars) to disease progression.	Difficulty of designing studies to limit variation between samples [24].	Glutathione metabolism in ferroptosis evasion [25]

**Table 2:** Original TNBCtype classes.

	Subtype	Pathways
Basal-like	Basal-like 1 (BL1)	Proliferative gene pathways (cell cycle, DNA replication), usually associated with high Ki-67
	Basal-like 2 (BL2)	Growth factor genes
	Immunomodulatory (IM) *	Immune cell signaling
Mesenchymal	Mesenchymal-like (M)	Cell motility, cell differentiation, WNT, ALK, extracellular matrix
	Mesenchymal stem-like (MSL) **	Growth factor and epithelial-to-mesenchymal transition
Luminal	Luminal androgen receptor (LAR)	Androgen/estrogen metabolism, steroid biosynthesis, porphyrin metabolism

* The IM subtype has since been identified to be immune infiltrate within the tumor on validation with laser microdissection methods [15]. ** The MSL subtype has been identified to be tumor-associated stromal cells via validation with laser microdissection methods [15].

**Table 3:** TNBCtype-4 targets.

Subtype	Gene Findings	Citation
BL1	Upregulated DNA/RNA synthesis, cell division, and nuclear export	[46]
BL2	Upregulated extracellular matrix, collagen, cell junction, and cell membrane components	[46]
M	Lowly expresses PD-L1, making immunotherapy less effective	[47]
LAR	PRC-2, enhances chemotherapy response Genetic dependency on CCND1 GPX4, can be inhibited to cause ferroptosis Activating mutation in PIK3CA	[25,44,45,47]

**Table 4:** Potential markers of TNBC identified by -omics techniques.

Markers	Techniques	Function	Validation Status	Citations
MIF	RNA-seq, scRNA-seq, spatial transcriptomics	Regulates glucocorticoid immunosuppression, mediating cell survival.	-omics-identified	[16,17,18,19]
CXCL13	scRNA-seq	Expressed in T cells to induce proinflammatory signaling in macrophages.	Pre-clinical validation	[65,76]
CD73/OTUD4	Proteomics and spatial transcriptomics	CD73 stabilizes OTUD4, causing accumulation and immunosuppression.	Pre-clinical validation	[11,12]
VIM	scRNA-seq	Intermediate filament protein found in mesenchymal cells. Drives epithelial to mesenchymal transition.	Pre-clinical validation	[68,69,70,71]
CALD1	scRNA-seq	Actin-binding protein involved in cell motility. Drives epithelial to mesenchymal transition.	-omics-identified	[71,72,73]
